# Anti-inflammatory and Analgesic Effects of *Polygonum orientale* L. Extracts

**DOI:** 10.3389/fphar.2017.00562

**Published:** 2017-08-30

**Authors:** Kai-Jun Gou, Rui Zeng, Yan Dong, Qi-Qi Hu, Huang-Wan-Yin Hu, Katherine G. Maffucci, Qi-Ling Dou, Qing-Bo Yang, Xu-Hua Qin, Yan Qu

**Affiliations:** ^1^College of Pharmacy, Chengdu University of Traditional Chinese Medicine Chengdu, China; ^2^College of Pharmacy, Southwest University for Nationalities Chengdu, China; ^3^Affiliated Hospital of Chengdu University of Traditional Chinese Medicine Chengdu, China; ^4^Department of Chemistry, Stony Brook University, Stony Brook NY, United States; ^5^Guizhou Yibai Pharmaceutical Co., Ltd. Guizhou, China

**Keywords:** *Polygonum orientale* L., UPLC-Q-Exactive HRMS, anti-inflammatory effect, analgesic effect, inflammatory mediators

## Abstract

**Background and Purpose:** *Polygonum orientale* L. (family: Polygonaceae), named Hongcao in China, is a Traditional Chinese Medicinal and has long been used for rheumatic arthralgia and rheumatoid arthritis. However, no pharmacological and mechanism study to confirm these clinic effects have been published. In this investigation, the anti-inflammatory, analgesic effects and representative active ingredient compounds of *P. orientale* have been studied.

**Methods:** Dried small pieces of the stems and leaves of *P. orientale* were decocted with water and partitioned successively to obtain ethyl acetate and ethyl ether extract of *P. orientale* (POEa and POEe). Chemical compositions of them were analyzed by UPLC-Q-Exactive HRMS. Anti-inflammatory and analgesic effects of POEa and POEe were evaluated using xylene induced ear edema, carrageenan induced paw edema, Freunds’ complete adjuvant induced arthritis, and formaldehyde induced pain in rat. Their mechanisms of anti-inflammatory and analgesic effects were also studied via assays of TNF-α, IL-1β, IL-6, and PGE2 in serum.

**Results:** UPLC-Q-Exactive HRMS analysis showed that POEa and POEe mainly contained flavonoids including orientin, isoorientin, vitexin, luteolin, and quercetin. Furthermore, anti-inflammatory effects of POEa and POEe were evident in xylene induced ear edema. The paw edema in Freund’s complete adjuvant and carrageenan were significantly (*P* < 0.05, 0.01) inhibited by POEa (5, 7.5 g/kg). POEe (7.5 g/kg) was significantly (*P* < 0.05, 0.01) inhibited Freunds’ complete adjuvant induced paw edema and cotton pellet induced granuloma formation. Similarly, POEe significantly (*P* < 0.05, 0.01) inhibited the pain sensation in acetic acid induced writhing test. POEa (5, 7.5 g/kg) significantly (*P* < 0.05, 0.01) inhibited formaldehyde induced pain in both phases. POEa (7.5 g/kg) markedly (*P* < 0.05) prolonged the latency period of hot plate test after 30 and 60 min. The concentrations of TNF-α, IL-1β, IL-6, and PGE2 were significantly (*P* < 0.01) decreased by POEa (3.75, 5 g/kg).

**Conclusion:** POEa and POEe have anti-inflammatory and analgesic effects, which was mainly relevant to the presence of flavonoids, including orientin, isoorientin, vitexin, luteolin, and quercetin. The mechanism of anti-inflammatory and analgesic effects of POEa may be to decrease the concentrations of TNF-α, IL-1β, IL-6, and PGE2 in serum.

## Introduction

Inflammation is a protective response that produces a range of inflammatory mediators to irritation, infection, or tissue injury to eradicate the irritant or microbe and promote tissue repair (Sherwood and Toliver-Kinsky, 2004; Ricciotti and FitzGerald, 2011; Sajid et al., 2017). At the same time, it can cause different degrees of injury lasting beyond the acute response such as allergic reactions, edema, effusion, and scarring. At present, inflammatory diseases including arthritis, cardiovascular disease, diabetes mellitus, and so on have become major health concerns around the world, resulting in increased mortalities each year. NSAIDs, such as opioids and glucocorticoids, are commonly used to treat inflammation and related diseases. However, these drugs also exhibit side effects in the treatment of inflammation, such as gastric irritation, ulcers, hepatotoxicity, and renal failure with chronic administration (Kunanusorn et al., 2009). The study has been confirmed, the antioxidant effects of herbal extracts have been suggested to play a protective role in the management and prevention of oxidative stress and inflammatory-related diseases (Menghini et al., 2016; Ferrante et al., 2017; Locatelli et al., 2017). So, many medical practitioners acknowledge the need for research into medicinal plants to ameliorate these harmful side effects without compromising efficacy (Junaid et al., 2011). It is imperative to strengthen exploration to discover new anti-inflammatory drugs from natural plant medicines. Traditional Chinese folk medicine has utilized plants as medicine for 1000s of years. Thus, it is sensible to further investigate such herbs.

*Polygonum orientale* L. (Polygonaceae) is an annual well-known Chinese herb which can be found throughout many regions of China, especially the tropical zones of the southwest and southeast (Wang et al., 2010). It can be found in the vicinity of most bodies of water and in wetlands (Yang et al., 2008; Feng et al., 2016). *P. orientale* has the ability to dispel wind and dampness, promote blood circulation, and relieve pain (The Compilation Group of Chinese Herbal Medicine, 1996; State Administration of Traditional Chinese Medicine of People’s Republic of China, 1998). It has been officially listed in the local standard of traditional materia medica of Guizhou province, where the local communities use its stem and leaf for the treatment of rheumatoid arthritis and coronary heart disease (Drug Administration of Guizhou Province, 2003). There are limited modern pharmacological studies and show that *P. orientale* has antioxidant, anti-inflammatory, analgesic, anti-myocardial ischemic, and vasodilator activities (Liang et al., 2013, 2014). There is also evidence that *Fructus polygoni orientalis* (the fruit of *P. orientale*) has the ability to eliminate indigestion and relieve pain (Zhai et al., 2006). Some previous investigations showed water extract of *P. orientale* mainly contained flavonoids including isoorientin, orientin, vitexin, and quercetin (Huang et al., 2014), and these components all have anti-inflammatory and analgesic effects (Da Silva et al., 2010; Gorzalczany et al., 2011; Napimoga et al., 2013; Robbins et al., 2014). However, the mechanisms of anti-inflammatory and analgesic effects are unclear. As such, the main effective parts, active components, and mechanisms of anti-inflammatory and analgesic effects of *P. orientale* also need to be further studied.

Earlier pharmacological studies have shown that flavonoids, chemical constituents of traditional Chinese medicine, have anti-inflammatory and analgesic effects. At the same time, they played anti-inflammatory effects through other pathways and regulation of inflammatory mediators such as IL-1β, IL-6, and TNF-α and their receptors (Li et al., 2015; Yasui et al., 2015). In this study, most of the flavonoids were extracted by systematic solvent method. Then the components of POEa and POEe were qualitatively and quantitatively analyzed by UPLC-Q-Exactive HRMS. This study investigated the anti-inflammatory effects of POEa and POEe using xylene induced ear edema, carrageenan induced paw edema, cotton pellet induced granuloma, and FCA induced arthritis model. Their analgesic effects were evaluated by acetic acid induced writhing test, hot plate test, and formaldehyde induced pain test. Finally, the levels of inflammatory cytokines in serum *in vivo* were detected by ELISA to study mechanisms of anti-inflammatory and analgesic effects of POEa and POEe. So, the aim of this study is to provide an experimental basis for clinical application and further development of *P. orientale* and also lay the foundation for further research on the treatment of rheumatoid arthritis.

## Materials and Methods

### Chemicals

All the standard reference substances such as orientin, orientin, vitexin, luteolin were purchased from Chengdu Pusi Biological Technology Co., Ltd (Sichuan, China). ELISA kits for TNF-α, IL-1β, IL-6, and PGE2 were provided from Nanjing Jiancheng Bioengineering Institute (Jiangsu, China). The standard drugs such as Dex and Tra were purchased from Xianju Pharmaceutical Co., Ltd (Zhejiang, China) and Stone Pharmaceutical Group Ouyi Pharmaceutical Co., Ltd (Beijing, China) respectively. Carrageenan and FCA were purchased from Sigma-Aldrich (United States). Chromatography of methanol and acetonitrile were purchased from Thermo Fisher Scientific Co., Ltd. (Shanghai, China). Cotton pellet, petroleum ether, ethyl ether, ethyl acetate, xylene, acetic acid, formaldehyde, formic acid, and the other chemicals were of analytical grade and were purchased from Chengdu Kelon Chemical Reagent Factory (Sichuan, China).

### Plant Collection

*Polygonum orientale* was obtained from Guizhou Yibai Pharmaceutical Co., Ltd. (Guizhou, China) in April 2016. The specimen was identified by Professor Lin-Fang Huang, Institute of Medicinal Plant Development, Chinese Academy of Medical Sciences and Peking Union Medical College. A voucher specimen was deposited with the number YB5892 in the Herbarium of the Guizhou Yibai Pharmaceutical Co., Ltd.

### Preparation of Extracts

Small pieces of dried *P. orientale* (480 g) were soaked with distilled water for 30 min and extracted three times (30 min, per time) with 10 times the amount of distilled water (v/m = 10 ml/g, about 4.8 L). The water decoction from the combined extractions was concentrated to about 200 ml using a rotary evaporator at 80°C. The above concentrated decoction was extracted and partition by a separatory funnel (extraction solvent: petroleum ether, ethyl ether, ethyl acetate), and each part was extracted until colorless. Petroleum ether extraction was discarded, leaving POEa and POEe. Finally, POEa and POEe were dried by rotary evaporator (Shanghai Yarong biochemical equipment factory, China) and stored in a 4°C. The percent yield of POEa and POEe were 0.73, 0.61%, respectively.

### UPLC-Q-Exactive HRMS Analysis of Extracts

The components of POEa and POEe were analyzed by UPLC-Q-Exactive HRMS [Ultimate 3000 (Dionex, United States)]. The chromatographic conditions were as follows: UPLC column specification was an ACQUITY UPLC BEH C18 2.1 mm × 100 mm, 1.7 μm (Waters, United States) at 35°C. The gradient elution was carried out with aqueous formic acid 0.1% (v) as mobile phase A and acetonitrile as phase B at a flow rate of 0.3 mL/min (0–30 min, 13–21% B; 30–35 min, 21–13% B). The injection volume was 2 μl, the injection temperature was 15°C, and the detection wavelength was 350 nm.

Tandem mass spectrometry was performed with a Q Exactive Orbitrap MS (Thermo Fisher, United States). Mass spectrometry conditions: negative ion detection mode; spray voltage, 2.00 kv; sheath gas pressure 206.8 KPa; auxiliary gas volume flow and temperature, 10 L/min and 350°C; ion transport tube temperature, 320°C; scanning modes: full MS (resolution 70000) and MS/MS (resolution 17500, normalized collision energy 35 eV, stepped normalized collision energy 30 and 40 eV) and scan range, m/z 80–1200.

### Drug Treatments

Distilled water with 0.1% (v/v) Tween-80 was used to dispense the extract and standard drug in the study. The animals received treatments as follows: groups I–VIII: solvent control (0.1% Tween-80); POEa (Extract 3.75, 5, and 7.5 g/kg); POEe (Extract 3.75, 5, and 7.5 g/kg); standard drugs (Dex: dexamethasone acetate 0.005 g/kg; Tra: tramadol hydrochloride 0.04 g/kg) (Xue et al., 2016).

The terms POEa and POEe stand for *P. orientale* ethyl acetate extract and *P. orientale* ethyl ether extract, respectively. All the treatments involved intragastric administration (i.g.) to the animals in weight-based dosages. All doses of POEa and POEe are converted into rat or mouse dose of 3.75–7.5 g/kg based on the adult daily dosage of 25–50 g of “The National Herbal Medicine Compilation” treatment of rheumatoid arthritis (The Compilation Group of Chinese Herbal Medicine, 1996; Liang et al., 2014).

### Animals

SPF grade Kunming male mice (18–22 g) and Sprague-Dawley male rats (180–220 g) were obtained from Chengdu Dasuo Experimental Animal Co., Ltd (Sichuan, China). Animals were kept at 24 ± 3°C with a 12 h dark/light cycle in a standard laboratory and fed a diet in accordance with standard laboratory protocols. Animals required adaptive feeding for at least a week, and fasting for 12 h before the experiments. The experimental protocol followed the principles and guidelines recommended by the Chinese Society of Experimental Animals and was approved by the local Ethical Committee of the Institute.

### Determination of Anti-inflammatory Activity

#### Xylene Induced Ear Edema

Mouse ear edema was induced using xylene solvent as previously described (Hosseinzadeh and Younesi, 2002). Kunming male mice (total = 64) were randomly divided into eight groups of eight, each according to their body weight: groups I–VIII. Dex was used as the standard anti-inflammatory drug. Mice were treated with 0.2 ml/10 g intragastric (i.g.) administered once daily for 3 days. After the administration of extracts or standard drug for 30 min, each mouse was externally treated with a test substance at 0.05 ml 100% xylene solvent on surface of the left ear, and the right ear served as the control. After 30 min of xylene application, the mice were euthanized under ketamine anesthesia. Circular sections (diameter: 6 mm) of both ears of each mouse were removed and weighed using an electronic analytical balance (Shanghai Shun Yu Hengping Science Instrument Co., Ltd., China) with 0.1 mg precision to calculate the inhibition of ear edema.

Ear swelling = Weight of left ear – Weight of right ear;

Percent inhibition (%) = [Ear swelling (control) – Ear swelling (test)] × 100/Ear swelling (control).

#### Cotton Pellet Induced Granuloma

Mouse cotton pellet induced granuloma model was produced according to previous experimental method (Liang et al., 2014; Sengar et al., 2015). Grouping of Kunming male mice (total = 64, 8 per group) are the same as “2.6.1” item. After intraperitoneal (i.p.) injection and anesthesia under 1% pentobarbital sodium (5 ml/kg), a 1 cm long lesion was introduced on the left side of the abdomen and disinfected with iodophor. A 10 mg sterilized dry cotton pellet (10 g/L ampicillin solution soaked and dried) was implanted into the subcutaneous tissue of mouse abdomen. From the day of surgery, animals received the test drugs once daily for 7 days and were euthanized under ether anesthesia on the 8th day. Pellets were taken out (granuloma tissue was removed), dried, weighed in an electronic analytical balance, and used to calculate percent inhibition.

Percent inhibition (%) = [Granuloma mass (control) – Granuloma mass (test)] × 100/Granuloma mass (control).

#### Carrageenan Induced Paw Edema

Anti-inflammatory activities of POEa and POEe were studied by Carrageenan induced paw edema model in mice (Muhammad et al., 2012). Grouping and administration method of Kunming male mice (total = 64, 8 per group) are the same as “2.6.1” item. After 30 min of the last treatment, the left hind paw of each mouse was injected subcutaneously with 50 μl carrageenan (1%, w/v saline) (Passos et al., 2007; Azevedo et al., 2016). Paw volume was measured using a digital plethysmometer (Ugo Basile, Co., Italy) at 0th, 1st, 2nd, 3rd, 4th, and 5th h after treatment (Vittalrao et al., 2011). After 5 h, the animals were euthanized. Their right and left paws were removed and weighed in an electronic analytical balance, and percent inhibition of paw edema was calculated (Rodrigues et al., 2016).

Inhibition (%) = [(M_T_ – M_0_) control – (M_T_ – M_0_) treated group] × 100/(M_T_ – M_0_) control. Where M_0_ = the basal paw weight (g), M_T_ = the difference between the paw weight (g) after inflammatory injury.

#### Freunds’ Complete Adjuvant Induced Arthritis

This inflammation model was induced by intradermal injection of 100 μl of FCA into a foot pad of the left hind paw of each rat according to previously experimental method (Latha et al., 1998). Sprague-Dawley male rats (total = 64, 8 per group) were used in this experiment, grouping of them remains the same as “2.6.1” item, and the rats were treated with 10 ml/kg i.g. On second day after FCA immunization, the rats were treated once daily for 28 days. The paw edema volume in each rat was periodically examined from the ankle via plethysmometer on 0th, 7th, 14th, 21st, and 28th days after administration of the drugs (Sengar et al., 2015).

### Determination of Analgesic Activity

#### Acetic Acid Induced Writhing Test

The analgesic activities of POEa and POEe can be determined according to the experimental method previously recorded (Zhu et al., 2011; Khan et al., 2013). Tra was used as the standard analgesic drug, and other grouping and administration method of Kunming male mice (total = 48, 8 per group) were the same as “2.6.1” item. After 60 min of administration, 0.6% acetic acid (0.2 ml/10 g) was intraperitoneally injected into mice to induce writhing. The number of writhes of each mouse in a transparent observation box was counted during 20 min after the acetic acid administration and calculated percent inhibition as follows:

Inhibition (%) = [Number of writhings (control) – Number of writhings (test)] × 100/Number of writhings (control).

#### Hot Plate Test

Kunming male mice (total = 48, 8 per group) were used to perform hot plate test (Franzotti et al., 2000). Before the experiment, the mice were subjected to pre-testing on a hot plate (Harvard Apparatus, Ltd, United Kingdom) maintained at 55 ± 0.1°C. The latency of the mice licking hind legs was the pain threshold. Only mice that showed initial nociceptive responses between 5 and 30 s were selected for the experiment. In order to avoid tissue damage, cut-off time of 60 s was set for all animals and room temperature was kept around 15°C. Grouping and administration method of mice are the same as “2.7.1” item. The latency time was recorded for each group at 0, 30, 60, 90, and 120 min after administration of the drugs.

#### Formaldehyde Induced Pain

The formaldehyde induced pain model was established according to previous experimental method with slight modifications (Mo et al., 2013). Sprague-Dawley male rats (total = 64, 8 per group) were used in this experiment, grouping of them are the same as “2.7.1” item, and these rats were treated with 1 ml/100 g i.g, administered once daily for 3 days. Thirty min after administration of the drug, 50 μl of 2% formaldehyde solution was injected subcutaneously into the rat left hind paw. Immediately, spontaneous nociceptive behaviors were determined by measuring number and duration of paw licking (Suzuki et al., 2009; Marahel and Umesha, 2016) were recorded from 0 to 10 min (First-phase, neurogenic) and from 11 to 60 min (Second-phase, inflammatory) after the formaldehyde injected paw (Liu et al., 2008). Percent inhibition of number and duration of licking were calculated as follows:

Inhibition (%) = [Number/duration of paw licking (control) – Number/duration of paw licking (test)] × 100/Number/duration of paw licking (control).

### Serum TNF-α, IL-1β, IL-6, and PGE2 Assays

After 1 h of injection of formaldehyde solution, all Sprague-Dawley male rats (total = 64, 8 per group) were anesthetized with an intraperitoneal injection of 10% chloral hydrate solution urethane (3 ml/kg). Blood samples were collected from the femoral artery and allowed to clot for 1 h. Serum was obtain by centrifuging at 3000 r⋅min^-1^ for 10 min to and stored at 20°C. Finally, levels of TNF-α, IL-1β, IL-6, and PGE2 in serum *in vivo* were determined using ELISA kits according to the manufacturer’s instructions (Cai et al., 2009; Han et al., 2016).

### Statistical Analysis

All data were analyzed and calculated by SPSS 21.0 software and expressed as mean ± SD. Significance was determined by using one-way ANOVA followed by Dunnett’s test or Tukey’s test. In all cases, differences of *P* < 0.05 were considered as statistically significant. The data were analyzed using software Graph Pad prism version 6.07.

## Results

### UPLC-Q-Exactive HRMS Analysis of Extracts

**FIGURE 1:**
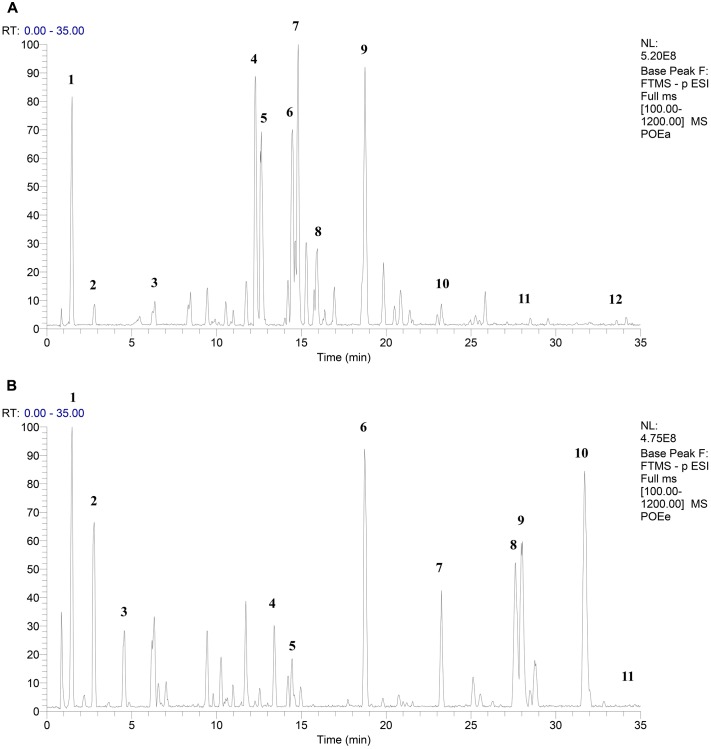
UPLC-Q-Exactive HRMS base peak chromatogram of POEa **(A)** and POEe **(B)**.

Combined with the relevant literature and UPLC-Q-Exactive HRMS analysis, it was determined that POEa contained gallic acid, protocatechuic acid, catechol, isoorientin, orientin, vitexin, isovitexin, luteolin-7-*O*-β-D-glucoside, quercitrin-3-*O*-α-L-rhamnoside, kaempferide-3-*O*-α-L-rhamnoside, luteolin, and apigenin. POEe was found to contain gallic acid, protocatechuic acid, catechol, taxifolin, vitexin, quercitrin-3-*O*-α-L -rhamnoside, kaempferide-3-*O*-α-L-rhamnoside, quercetin, luteolin, isorhamnetin, and apigenin (Wang et al., 2007; Huang et al., 2011; Xu and Chen, 2012; Ren and Zhang, 2013; Chen et al., 2014; Sheng et al., 2014; Liu et al., 2015; Gu and Yang, 2016). The main elements of POEa and POEe were flavonoids, including the high concentrations of orientin, isoorientin, vitexin, luteolin, and quercetin. Results are shown in **Figures 1A,B** and **Table 1**.

**Table 1:** Analysis of POEa and POEe by UPLC-Q-Exactive Orbitrap-MS.

Comp.no. [retention] [time (min)]		Molecular formula	Experimental m/z [M–H]^-^	MS^2^	RDB	Delta (ppm)	Compounds	Classification	Reference
	
POEa	POEe								
1 (1.48)	1 (1.48)	C_7_H_6_O_5_	169.0131	125.0231[M-H-CO_2_]**^-^**	5.5	-0.176	Gallic acid	Phenolic	Liu et al., 2015
2 (2.82)	2 (2.79)	C_7_H_6_O_4_	153.0181	109.0281[M-H-CO_2_]**^-^**	5.5	-0.622	Protocatechuic acid		Huang et al., 2011
3 (6.36)	3 (6.33)	C_15_H_14_O_6_	289.0717	245.0816[M-H-CO_2_]**^-^**; 203.0706[M-H-CO_2_-C_2_H_2_O]**^-^**; 125.0231[M-HC_6_H_6_O_2_-C_2_H_4_O]**^-^**	9.5	3.651	Catechol	Flavonoids	Chen et al., 2014
4 (12.28)		C_21_H_20_O_11_	447.0930	357.0617[M-H-C_3_H_6_O_3_]**^-^**; 327.0511[M-H-C_4_H_8_O_4_]**^-^**	12.5	1.727	Isoorientin		Standard
5 (12.64)		C_21_H_20_O_11_	447.0932	357.0617[M-H-C_3_H_6_O_3_]**^-^**; 327.0511[M-H-C_4_H_8_O_4_]**^-^**	12.5	2.063	Orientin		Standard
	4 (13.40)	C_15_H_12_O_7_	303.0510	285.0404[M-H-H_2_O]**^-^**; 175.0391[M-H-H_2_O-C_3_H_4_OCO_2_]**^-^**	10.5	3.402	Taxifolin		Huang et al., 2011
6 (14.48)	5 (14.48)	C_21_H_20_O_10_	431.0980	341.0667[M-H-C_3_H_6_O_3_]**^-^**; 311.0562[M-H-C_4_H_8_O_4_]**^-^**	12.5	1.732	Vitexin		Standard
7 (14.81)		C_21_H_20_O_10_	431.0978	341.0667[M-H-C_3_H_6_O_3_]**^-^**; 311.0562[M-H-C_4_H_8_O_4_]**^-^**	12.5	1.454	Isovitexin		Sheng et al., 2014
8 (15.95)		C_21_H_20_O_11_	447.0932	285.0404[M-H-C_6_H_10_O_5_]**^-^**	12.5	2.197	Luteolin-7-*O*-β-D-glucoside		Xu and Chen, 2012
9 (18.76)	6 (18.76)	C_21_H_20_O_11_	447.0932	301.0276[M-H-C_6_H_10_O_4_]**^-^**	12.5	2.197	Quercitrin-3-*O*-α-L-rhamnoside		Gu and Yang, 2016
10 (23.27)	7 (23.27)	C_21_H_20_O_10_	431.0981	285.0403[M-H-C_6_H_10_O_4_]**^-^**	12.5	1.872	Kaempferide-3-*O*-α-L-rhamnoside		Wang et al., 2007
	8 (27.64)	C_15_H_10_O_7_	301.0352	178.9976[M-H-C_7_H_6_O_2_]**^-^**; 151.0025[M-H-C_7_H_6_O_2_-CO]**^-^**	11.5	2.926	Quercetin		Ren and Zhang, 2013
11 (28.01)	9 (28.03)	C_15_H_10_O_6_	285.0404	285.0404[M-H]**^-^**	11.5	3.668	Luteolin		Standard
	10 (31.70)	C_16_H_12_O_7_	315.0509	300.0274[M-CH_3_]**^-^**	11.5	3.082	Isorhamnetin		Ren and Zhang, 2013
12 (34.70)	11 (34.67)	C_15_H_10_O_5_	269.0455	269.0455[M-H]**^-^**	11.5	3.903	Apigenin		Standard


### Anti-inflammatory Activity

#### Xylene Induced Ear Edema

**FIGURE 2:**
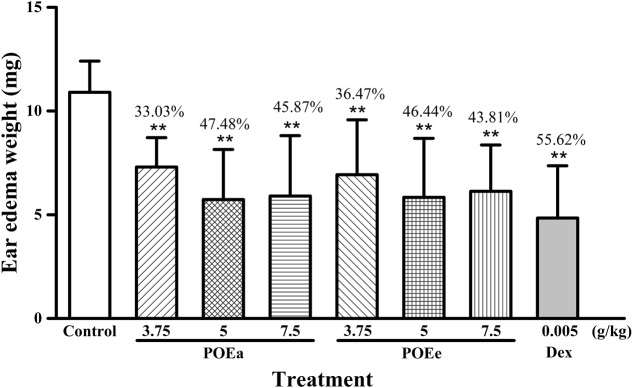
POEa = *P. orientale* ethyl acetate extract; POEe = *P. orientale* ethyl ether extract. Ear edema weight (mg) is presented as mean ± SD (*n* = 8). ^∗^*P* < 0.05, ^∗∗^*P* < 0.01 versus vehicle treated control using one-way ANOVA followed by Tukey’s *post hoc* multiple-comparison test.

The results of xylene induced ear edema are shown in **Figure 2**. Compared to the control group, all doses (3.75, 5, and 7.5 g/kg) of POEa and POEe significantly (*P* < 0.01) inhibited ear edema. Dexamethasone acetate (Dex: 0.005 g/kg) had a significant (*P* < 0.01) inhibitory effect on the auricle swelling of mice induced by xylene.

#### Cotton Pellet Induced Granuloma

**FIGURE 3:**
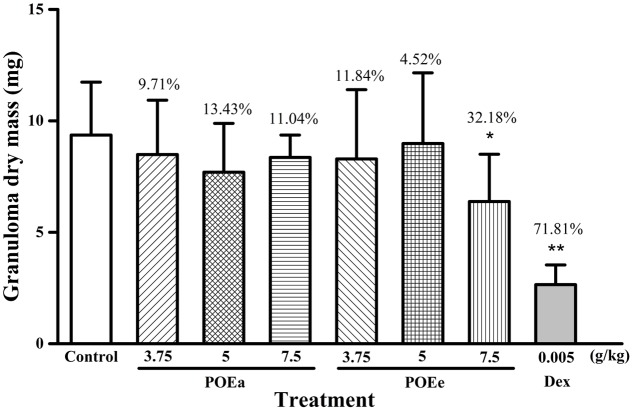
POEa = *P. orientale* ethyl acetate extract; POEe = *P. orientale* ethyl ether extract. Granuloma dry mass (mg) is presented as mean ± SD (*n* = 8). ^∗^*P* < 0.05, ^∗∗^*P* < 0.01 versus vehicle treated control using one-way ANOVA followed by Tukey’s *post hoc* multiple-comparison test.

The experimental results of cotton pellet induced granuloma in mice are shown in **Figure 3**. Compared with the control group, POEe (7.5 g/kg) had a significant (*P* < 0.05) inhibitory effect against cotton pellet induced granuloma formation, whereas POEa had no significant inhibitory effects. Dex (0.005 g/kg) also showed a significant (*P* < 0.01) inhibitory effect in granuloma formation.

#### Carrageenan Induced Paw Edema

**FIGURE 4:**
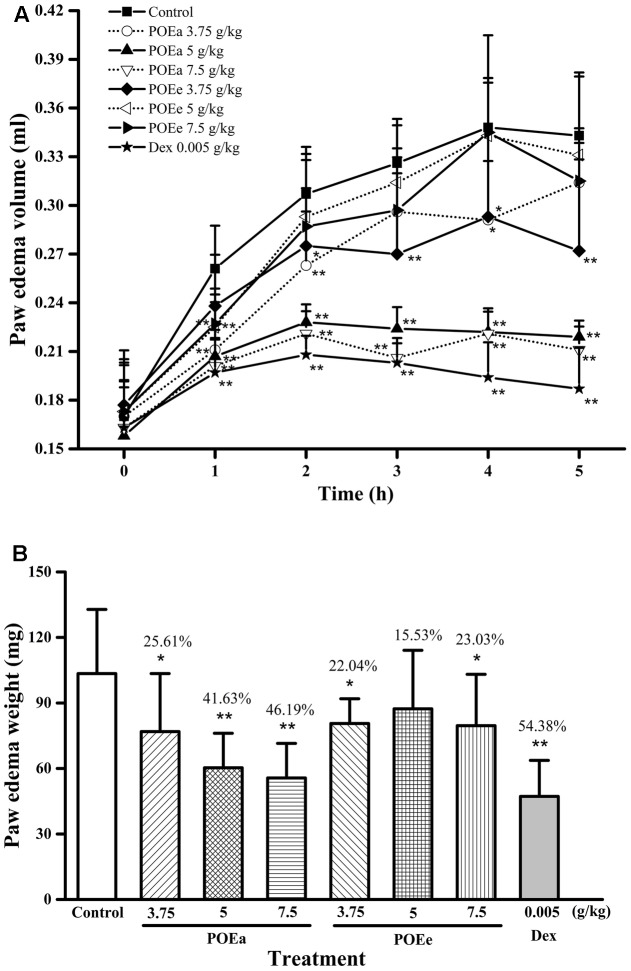
POEa = *P. orientale* ethyl acetate extract; POEe = *P. orientale* ethyl ether extract. Paw edema volume (ml) **(A)** and weight (mg) **(B)** are presented as mean ± SD (*n* = 8). ^∗^*P* < 0.05, ^∗∗^*P* < 0.01 versus vehicle treated control using one-way ANOVA followed by Tukey’s *post hoc* multiple-comparison test.

The effects of POEa and POEe on carrageenan induced paws edema volume in mice are statistically significant in **Figure 4A**. Compared with the control group, POEa (3.75, 5, and 7.5 g/kg) were significantly (*P* < 0.05, 0.01) inhibited in a dose-dependent manner after 1st, 2nd, 3rd, 4th, and 5th administrations. POEe (5, 7.5 g/kg) showed significant (*P* < 0.01) reduction in paw edema volume at 1st h and had no significant reduction effects after the 1 h time point. POEe (3.75 g/kg) showed significant (*P* < 0.05, 0.01) reduction in paw edema volume after other time points except 1 h. Dex (0.005 g/kg) exhibited significant (*P* < 0.01) inhibitory effects on carrageenan induced paw edema throughout the experiment.

Similarly, the results of the weight of the paws in mice are shown in **Figure 4B**. Compared to control group, POEa (3.75, 5, and 7.5 g/kg) significantly (*P* < 0.05, 0.01) reduced paw weight. POEe (3.75, 7.5 g/kg) caused significant (*P* < 0.05) reduction in weight of paws. Dex (0.005 g/kg) also exhibited significant (*P* < 0.01) inhibitory effects on carrageenan induced paw edema.

#### Freunds’ Complete Adjuvant Induced Arthritis

**FIGURE 5:**
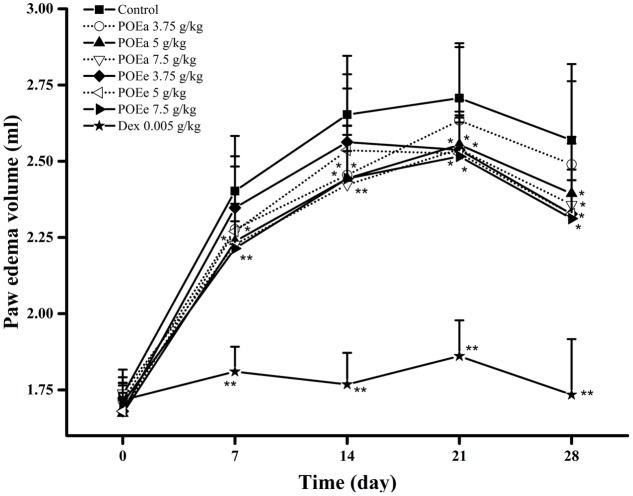
POEa = *P. orientale* ethyl acetate extract; POEe = *P. orientale* ethyl ether extract. Paw edema volume (ml) is presented as mean ± SD (*n* = 8). ^∗^*P* < 0.05, ^∗∗^*P* < 0.01 versus vehicle treated control using one-way ANOVA followed by Tukey’s *post hoc* multiple-comparison test.

The anti-inflammatory effects of POEa and POEe from this model are demonstrated from the days 7 to 28 in **Figure 5**. Compared to the control group, adjuvant induced arthritis was significantly (*P* < 0.05, 0.01) reduced, indicated by paw volume decrease by treatment with POEa (5, 7.5 g/kg) and POEe (7.5 g/kg) after 7th, 14th, 21st, and 28th days. POEe (3.75, 5 g/kg) significantly (*P* < 0.05) reduced the paw edema volume after days 21 and 28. Dex (0.005 g/kg) also exhibited significant (*P* < 0.01) reduction in paw edema volume after 7th, 14th, 21st, and 28th days.

### Analgesic Activity

#### Acetic Acid Induced Writhing Test

**FIGURE 6:**
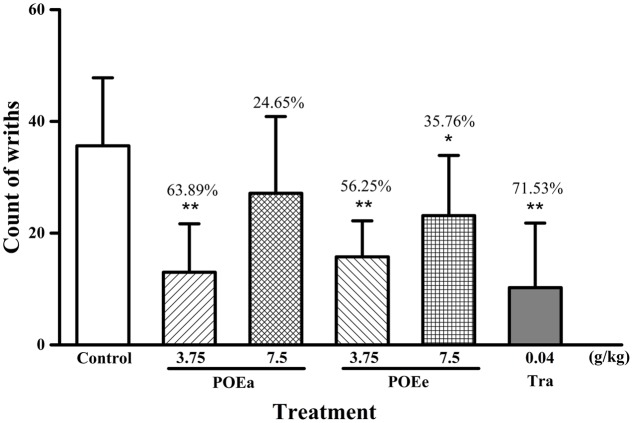
POEa = *P. orientale* ethyl acetate extract; POEe = *P. orientale* ethyl ether extract. Count of writhes are presented as mean ± SD (*n* = 8). ^∗^*P* < 0.05, ^∗∗^*P* < 0.01 versus vehicle treated control using one-way ANOVA followed by Tukey’s *post hoc* multiple-comparison test.

The numbers of writhes and percent inhibition of analgesia by acetic acid induced writhing in mice after administration of the drug are shown in **Figure 6**. Compared to control group, POEa (3.75 g/kg) significantly (*P* < 0.01) decreased writhing in mice. However, POEa (7.5 g/kg) showed no significant reduction in writhing. Varying doses (3.75, 7.5 g/kg) of POEe significantly (*P* < 0.05, 0.01) decreased writhing. Tramadol hydrochloride (Tra: 0.04 g/kg) also showed significant (*P* < 0.01) inhibition of analgesic activity.

#### Hot Plate Test

**FIGURE 7:**
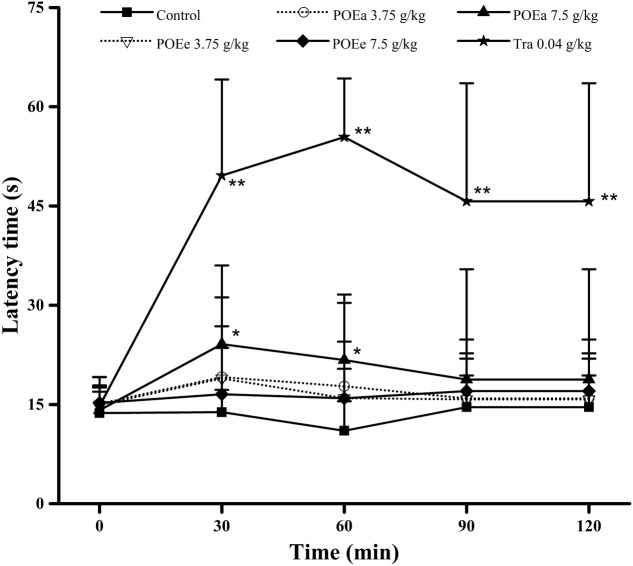
POEa = *P. orientale* ethyl acetate extract; POEe = *P. orientale* ethyl ether extract. Latency time (second) is presented as mean ± SD (*n* = 8). ^∗^*P* < 0.05, ^∗∗^*P* < 0.01 versus vehicle treated control using one-way ANOVA followed by Tukey’s *post hoc* multiple-comparison test.

The results of the hot plate test are presented in **Figure 7**. Compared with the control group, POEa (7.5 g/kg) markedly (*P* < 0.05) prolonged the latency period of mice during after 30 and 60 min. However, the pain threshold in mice wasn’t significantly prolonged after administration of POEe. Tra (0.04 g/kg) significantly (*P* < 0.01) prolonged the latency period of mice at all times.

#### Formaldehyde Induced Pain

**FIGURE 8:**
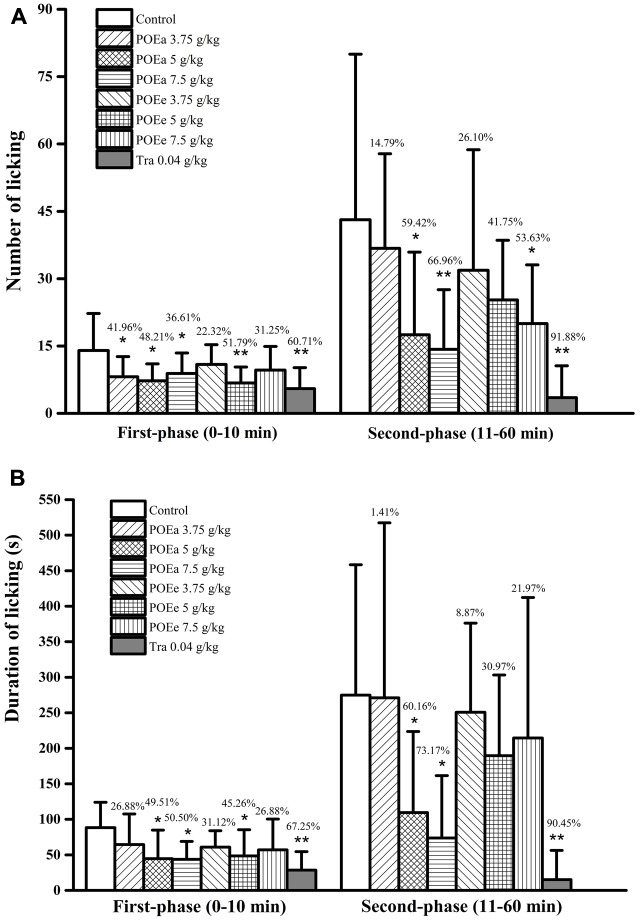
POEa = *P. orientale* ethyl acetate extract; POEe = *P. orientale* ethyl ether extract. Number of licking **(A)** and duration of licking **(B)** are presented as mean ± SD (*n* = 8). ^∗^*P* < 0.05, ^∗∗^*P* < 0.01 versus vehicle treated control using one-way ANOVA followed by Tukey’s *post hoc* multiple-comparison test.

Number and duration of paw licking after formaldehyde administration are shown in **Figures 8A,B**. Compared with the control group, POEa (5, 7.5 g/kg) significantly (*P* < 0.05, 0.01) reduced the number and duration of licking after formaldehyde induced pain in both phases. POEe used in this study exhibited a moderate level of analgesic effects in both phases. Tra (0.04 g/kg) had significant (*P* < 0.01) reduction on number and duration of licking after formaldehyde induced pain in both phases. In addition, there were no significant side effects on motor function in mice after the injection of saline or formaldehyde.

### Serum TNF-α, IL-1β, IL-6, and PGE2 Assays

**FIGURE 9:**
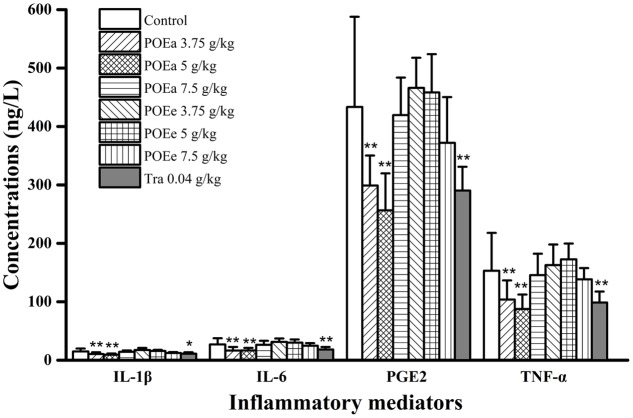
POEa = *P. orientale* ethyl acetate extract; POEe = *P. orientale* ethyl ether extract; interleukin-1β (IL-1β), interleukin-6 (IL-6), prostaglandin E2 (PGE2), tumor necrosis factor-α (TNF-α). Count of cells is presented as mean ± SD (*n* = 8). ^∗^*P* < 0.05, ^∗∗^*P* < 0.01 versus vehicle treated control using one-way ANOVA followed by Tukey’s *post hoc* multiple-comparison test.

The assessments of inflammatory mediators of formaldehyde induced pain in mice are shown in **Figure 9**. The concentrations of TNF-α, IL-1β, IL-6, and PGE2 in serum *in vivo* after administration of POEa (3.75, 5 g/kg) had significant (*P* < 0.01) reduction compared to the control group. However, POEa (7.5 g/kg) and POEe had no significant reduction in the concentrations of the inflammatory mediators. Tra (0.04 g/kg) demonstrated significant (*P* < 0.05, 0.01) reduction in the concentrations of all these inflammatory mediators.

**GRAPHICAL ABSTRACT:**
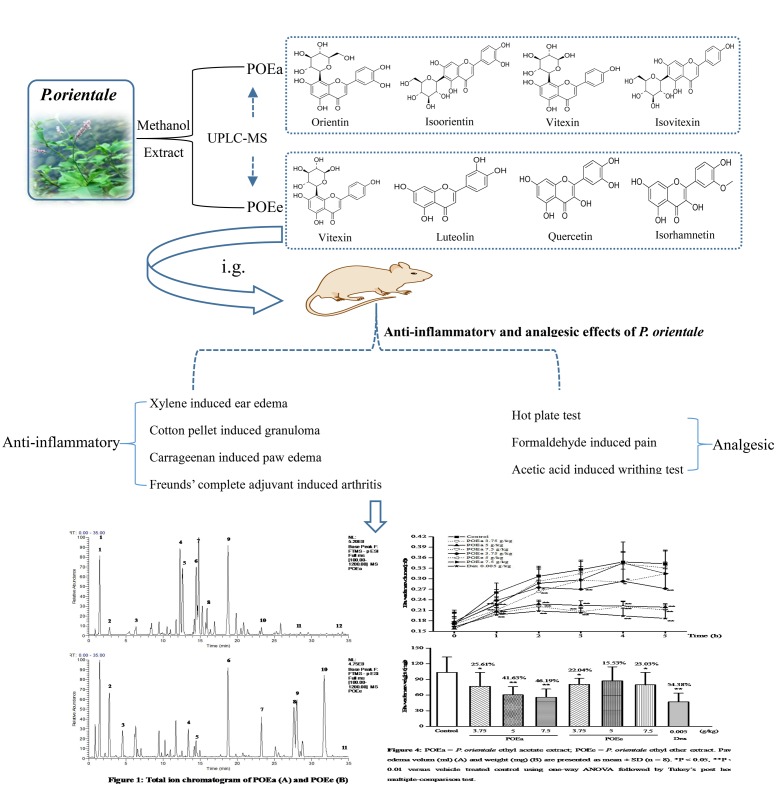
The experiment flow chart of anti-inflammatory and analgesic effects of *P. orientale* extracts.

## Discussion

*Polygonum orientale* is a folk herb of Guizhou Province in China that is frequently used to treat rheumatic arthralgia and rheumatoid arthritis. The anti-inflammatory and analgesic effects and mechanisms of POEa and POEe suggest that *P. orientale* should be further evaluated as a natural drug for the treatment of inflammatory diseases. By comparison of standard reference substances and analysis by UPLC-Q-Exactive HRMS, chromatographic peaks and ionic data of POEa and POEe suggest that the anti-inflammatory and analgesic effects of the two parts of *P. orientale* are due to flavonoids with anti-inflammatory and analgesic activities (Sayyah et al., 2004; Aquila et al., 2009). The experiments evaluating anti-inflammatory and analgesic effects suggest that the effectiveness of POEa is related to its relatively higher flavonoid contents (orientin, isoorientin, vitexin, and isovitexin), and POEe’s effectiveness is due to its high vitexin, luteolin, quercetin, and isorhamnetin flavonoid contents. Of course, in order to confirm the anti-inflammatory and analgesic active components of *P. orientale*, a more in-depth study of these monomeric compounds is necessary.

In this study, several animal models were used to evaluate anti-inflammatory and analgesic activities and possibly elucidate mechanisms of POEa and POEe. To assess the anti-inflammatory properties of POEa and POEe, four models of cotton pellet induced granuloma, xylene induced ear edema, carrageenan induced paw edema, and Freunds’ complete adjuvant induced arthritis were used. Xylene induced mouse ear edema is an acute inflammatory model – during inflammation or injury. Histamine, serotonin and bradykinin are the main inflammatory mediators contained in this model. Production of histamine is an inflammatory response and is also considered an immune response, and it can promote vasodilatation and increase permeability (Benly, 2015). POEa and POEe significantly inhibited ear edema induced by acetic acid solution. Results also indicate that POEa and POEe significantly inhibit histamine-induced inflammation in mice.

Cotton pellet induced granuloma is often used to assess the chronic inflammatory effects of drugs. The reduction in granuloma formation by a therapeutic agent of interest can reflect inhibition of chronic inflammation (Panthong et al., 2003). POEe (7.5 g/kg) was found to significantly inhibit granuloma formation. However, POEa and the lower dose of POEe did not demonstrate significant effects. It indicates that treatment of chronic inflammation with POEe requires high concentrations of the active compounds.

Carrageenan induced paw edema in mice is an inflammation model consisting of two phases. In the initial phase of inflammation (0–2 h), the main release of histamine and bradykinin promotes the development of paw edema through the expansion of blood vessels and increased permeability (Sengar et al., 2015). In the later phase of inflammation (2–6 h), the release of prostaglandins mediated by leukotriene and bradykinin is a key factor in maintaining inflammation (Marzouk et al., 2010). In this study, paw edema volume and weight in mice were significantly reduced after administration of POEa. The study also demonstrated that POEa administration had the ability to dampen inflammatory response in both phases, suggesting that POEa may inhibit the production of the above inflammatory mediators. POEe (5, 7.5 g/kg) administration demonstrated a reduction in paw edema volume in the initial phase of inflammation, whereas POEe (3.75 g/kg) showed significant reduction in paw edema volume in the later phase of inflammation. POEe (3.75, 7.5 g/kg) caused significant reduction in weight of paws. It suggests that the anti-inflammatory phases of POEe may be related to dosage of administration.

Freunds’ complete adjuvant induced arthritis model is used to discover and evaluate drugs for the treatment of chronic inflammatory conditions (Bendele et al., 1999). Freunds’ complete adjuvant induced arthritis assessment relies on the premise that high levels of proinflammatory cytokines, such as TNF-α and interleukin-1 (IL-1), lead to inflammation and bone erosion by inducing cartilage degeneration and osteoclast differentiation, resulting in the emergence of arthritis (Niki et al., 2004). In this study, both POEa and POEe (7.5 g/kg) showed significant inhibition of Freunds’ complete adjuvant induced arthritis in mice at all times. So, POEa and the higher dose of POEe significantly inhibited inflammation, suggesting a role in the inhibition of proinflammatory mediators.

To investigate analgesic effects, we used the acetic acid induced writhing test, hot plate test, and formaldehyde induced pain model. Acetic acid induced writhing model in mice is a reliable test model for assessment of the analgesic effects of therapeutic drugs (Popoola et al., 2016). The release of inflammatory mediators including AA, COX, prostaglandins, bradykinin, serotonin, and histamine by acetic acid administration result in pain and abdominal writhing through peripheral nociceptive sensitization (Hasan et al., 2014; Florentino et al., 2016). POEa (3.75 g/kg) and POEe were found to significantly reduce the number of writhings, indicating that POEa and POEe have analgesic activities against acetic acid-induced peripheral pain. However, the proposed mechanism of analgesic response requires further study.

The hot plate test is commonly used to test central-mediated anti-nociceptive effects and analgesic effect. The cyclooxygenase pathway promotes inflammatory pain via conversion of AA to PGE2 by COX-2, an important inflammatory mediator (Ricciotti and FitzGerald, 2011). In this study, POEa (7.5 g/kg) significantly increased latency time (during about 60 min in hot plate test), indicative of an anti-nociceptive effect (change if this is not accurate). The experiment suggests that the higher dose of POEa inhibited inflammation because of disruption of prostaglandin synthesis.

Formaldehyde induced pain model is a biphasic pain response. The glutamate mediators of formaldehyde-induced pain are thought to be involved in the first-phase of pain (0–5 min) (Kunanusorn et al., 2009). The second-phase (15–30 min) is an inflammatory pain response. Inflammatory mediators such as histamine, excitatory amino acids, and prostaglandins are involved in this phase. However, bradykinin is unique in that it affects both phases simultaneously (Rezayat et al., 1999). The results showed that POEa (5, 7.5 g/kg) demonstrated significant pain suppression in both phases of formaldehyde-induced pain. POEe exhibited moderate analgesic effects in both phases. These groundbreaking results indicate that POEa has analgesic effects on both the peripheral and central pain pathways simultaneously. The analgesic effects of POEa and POEe may depend on their anti-inflammatory activities.

Literature studies show the concentration of TNF-α increases when exogenous factors are damaged or attacked, which can cause inflammation and pain response. At the same time, it can promote the expression and synthesis of nitric oxide synthase (iNOS) and interleukin such as IL-6, IL-1β. So, TNF-α plays an important role in body defense, inflammation and immune response (Jan and Khan, 2016). POEa (3.75, 5 g/kg) significantly decreased the concentrations of TNF-α, IL-6, and IL-1β, indicating that POEa has potential anti-inflammatory activity, inhibiting the expression of iNOS and interleukin synthesis by blocking TNF-α pathway. In addition, PGE2 is an inflammatory mediator involved in the pathogenesis of many inflammatory diseases and acts as a pain-induced and perceptual medium (Davidson et al., 2014). The inhibition of PGE2 by POEa (3.75, 5 g/kg) indicates that this preparation may directly inhibit the activity of COX-2. However, there were no significant inhibitory effects of POEe on the release of TNF-α, IL-6, IL-1β, and PGE2.

In summary, POEa and POEe display significant inhibitory effects on acute and chronic inflammation, as well as analgesic effects on inflammatory pain. Compared with POEe, the anti-inflammatory analgesic activity of POEa is more significant. Given these exciting results, *P. orientale* has the potential to replace the side effects of non-steroidal drugs, as well as minimize the side effects of natural anti-inflammatory drugs or anti-inflammatory adjuvants. In addition, the study also provides a pharmacological basis for the folk treatment of rheumatic arthralgia and rheumatoid arthritis, and lays the foundation for further research on the treatment of rheumatoid arthritis, as well as an experimental precedent for comprehensive development and utilization of *P. orientale*.

## Conclusion

The results showed that POEa and POEe displayed excellent anti-inflammatory effects and also demonstrated analgesic effects, which may be related to alleviation of the inflammation and inflammatory mediators by flavonoids, including orientin, isoorientin, vitexin, luteolin, and quercetin. At the same time, POEa significantly reduced the concentrations of inflammatory mediators TNF-α, IL-1β, IL-6, and PGE2. Its anti-inflammatory and analgesic mechanisms are related to the reduction of these inflammatory markers. However, there was no significant relationship between the anti-inflammatory and analgesic effects of POEe and these inflammatory mediators. This study supported the fact the traditional application of *P. orientale* by the local communities for inflammatory disorders such as rheumatic arthralgia and rheumatoid arthritis.

## Ethics Statement

This study was conducted in strict accordance with the recommendations of the Guide lines for the Care and Use of Laboratory Animals of the Ministry of Science and Technology of China. The protocol was approved by the Committee on the Ethics of Animal Experiment of Chengdu University of Traditional Chinese Medicine, Sichuan Province, China (Approval ID: 2014DL-001).

## Author Contributions

YQ, X-HQ, and YD designed the research; RZ and YQ revised the manuscript; K-JG, Q-QH, and H-W-YH performed the experiments; KM helped in language revise and study design; Q-LD and Q-BY contributed reagents/materials/analysis tools; YQ, X-HQ, RZ, and K-JG analyzed the experimental data and K-JG wrote the paper. All authors read and approved the final manuscript.

## Conflict of Interest Statement

The authors declare that the research was conducted in the absence of any commercial or financial relationships that could be construed as a potential conflict of interest.

## Abbreviations

AA: arachidonic acid
COX: cyclooxygenase
Dex: dexamethasone acetate
ELISA: enzyme-linked immunosorbent assay
FCA: Freund’s complete adjuvant
IL-1β: interleukin-1 beta
IL-6: interleukin-6
iNOS: inducible nitric oxide synthase
NSAIDs: non-steroidal anti-inflammatory drugs
*P. orientale*: *Polygonum orientale* L.
PGE2: prostaglandin E2
POEa: ethyl acetate extract of *P. orientale*
POEe: ethyl ether extract of *P. orientale*
TNF-α: tumor necrosis factor-alpha
Tra: tramadol hydrochloride
UPLC-Q-Exactive HRMS: ultra performance liquid chromatography-Q-Exactive high resolution mass spectrometry
